# Draft genome sequence of *Enterobacter chengduensis* ECC445, isolated from fresh water in the West Indies

**DOI:** 10.1186/s12863-023-01116-7

**Published:** 2023-03-11

**Authors:** Matthieu Pot, Célia Ducat, Yann Reynaud, David Couvin, Séverine Ferdinand, Sébastien Breurec, Antoine Talarmin, Stéphanie Guyomard-Rabenirina

**Affiliations:** 1 Transmission, Reservoir and Diversity of Pathogens Unit, Pasteur Institute of Guadeloupe, Pointe-À-Pitre, France; 2 Faculty of Medicine Hyacinthe Bastaraud, University of the Antilles, Pointe-À-Pitre, France; 3 Centre for Clinical Investigation 1424, INSERM, Pointe-À-Pitre/Les Abymes, France; 4 Laboratory of Clinical Microbiology, University Hospital Centre of Guadeloupe, Pointe-À-Pitre/Les Abymes, France

**Keywords:** Caribbean, CRISPR spacer, *Enterobacter cloacae* complex, Environment, hsp60, Phylogeny, Typing

## Abstract

**Objectives:**

The *Enterobacter cloacae* complex is considered an important opportunistic pathogen. It comprises many members that remain difficult to delineate by phenotypic approaches. Despite its importance in human infection, there is a lack of information on associated members in other compartments. Here we report the first de novo assembled and annotated whole-genome sequence of a *E. chengduensis* strain isolated from the environment.

**Data description:**

ECC445 specimen was isolated in 2018 from a drinking water catchment point in Guadeloupe. It was clearly related to *E. chengduensis* species according to hsp60 typing and genomic comparison. Its whole-genome sequence is 5,211,280-bp long divided into 68 contigs, and presents a G + C content of 55.78%. This genome and associated datasets provided here will serve as a useful resource for further analyses of this rarely reported *Enterobacter* species.

## Objective

Members of the *E. cloacae* complex (ECC) are ubiquitous *Enterobacteriaceae* involved in various opportunistic infections in animals, humans and plants [1]. The taxonomy of this bacterial complex has been re-evaluated regularly [1–3]. There are to date 24 species and 22 *Enterobacter* genomospecies in this bacterial complex [3]. In parallel to whole-genome sequencing, different typing approaches have been proposed to help in identify them, such as the amplification of the partial gene coding for the heat-shock protein 60 (hsp60) [4].

The objective of this study is to report a draft genome sequence of an environmentally isolated *E. chengduensis*. We also provided a global overview of this species which is rarely described in the scientific literature. ECC445 strain is associated with a broader analysis of *Enterobacter* population diversity and was isolated from a water sample [5]. This sample was collected in June 2018 during prospective control of a catchment point in Petit-Bourg, Guadeloupe (French overseas territory; 16.188009 N, 61.659017 W). After serial dilutions, 100 mL were filtered (0.45 μm, Millipore, Guyancourt, France). The membrane was placed in 9 mL of buffered peptone water solution for overnight pre-enrichment at 37 °C. Then, 100 μL of broth were cultured aerobically onto chromogenic agar supplemented with ceftriaxone at 4 mg/L (CCA, CHROMagar, Paris, France).

ECC445 strain was isolated from this selective plate after overnight growth at 37 °C. It was initially identified as *E. cloacae* (97.7% ID) with the API-20E system (BioMérieux, Marcy-l'Étoile, France), and as *E. hormaechei* (99.9% ID) by matrix assisted laser desorption/ionization time–of–flight mass spectrometry (VITEK MS, BioMérieux, Marcy-l'Étoile, France) [6]. This strain was resistant to third-generation cephalosporins by chromosomal overproduction of AmpC (CA-SFM/EUCAST guideline 2018, disc diffusion method; DataFile 1; Table 1) [5, 7, 8].

**Table 1:** Overview of datafiles/datasets

Label	Name of DataFile /data set	File types (file extension)	Data repository and identifier (DOI or accession number)
DataFile 1	Table S1, ECC445 – Antibiotic resistance profile	MS Excel file (.xlsx)	Figshare, 10.6084/m9.figshare.21294888 [8]
DataFile 2	Script S1, Analysis script of *E. chengduensis*	Bash file (.bash)	Figshare, 10.6084/m9.figshare.22041197 [9]
DataFile 3	Table S2, ECC445 – Quast and short BUSCO summary	Normal text file (.txt)	Figshare, 10.6084/m9.figshare.21294882 [14]
DataFile 4	Table S3, *E. chengduensis* metadata from NCBI and quality control criteria	MS Excel file (.xlsx)	Figshare, 10.6084/m9.figshare.21294903 [15]
DataFile 5	Alignment S1, Roary – *E. chengduensis* core-gene alignment	Fasta file (.fasta)	Figshare, 10.6084/m9.figshare.22041257 [16]
DataFile 6	MLTree S1, IQ-TREE – Maximum likelihood phylogenetic tree of *E. chengduensis*	Newick tree format (.nhx)	Figshare, 10.6084/m9.figshare.22041293 [17]
DataFile 7	Figure S1, Maximum likelihood phylogenetic tree of *E. chengduensis*	Portable Document Format file (.pdf)	Figshare, 10.6084/m9.figshare.21294879 [18]
DataFile 8	Table S4, ECC445 – PathogenFinder summary	Normal text file (.txt)	Figshare, 10.6084/m9.figshare.21294915 [19]
DataFile 9	Table S5, ECC445 – CrisprCasFinder summary	Tab-separated values (.tsv)	Figshare, 10.6084/m9.figshare.21294924 [20]
DataFile 10	Table S6, CRISPR spacers comparison	MS Excel file (.xlsx)	Figshare, 10.6084/m9.figshare.21294933 [21]
DataSet 1	Partial hsp60 coding sequence of the ECC445 strain	Fasta file(.fasta)	NCBI Nucleotide, https://identifiers.org/nucleotide:OM687483 [10]
DataSet 2	Illumina reads of the ECC445 strain	SRA file (.sra)	NCBI Sequence Read Archive, https://identifiers.org/ncbi/insdc.sra:SRR16640525 [22]
DataSet 3	Annotated assembly of the ECC445 strain	Fasta file (.fasta)	NCBI Nucleotide, https://identifiers.org/nucleotide:JAKLRZ000000000 [23]

## Data description

Upon isolation and to better identify this bacterium, DNA analysis was conducted. Unless otherwise stated on the detailed methodology file, default parameters were used for all software tools (DataFile 2) [9]. DNA was extracted from a pure isolate with the QIAamp DNA minikit (Qiagen, Hilden, Germany). We conducted the hsp60 typing approach and obtained a 273-bp amplicon that was sequenced at Eurofins (Eurofins Genomic SAS, Les Ullis, France; DataSet 1) [4, 5, 10]. BLASTn submission to the NCBI nucleotide collection database indicated 100% coverage and a maximum percentage identity with the *E. chengduensis* reference (v2.11.0; 99.27%; CP043318) [11–13].

The second sequencing step was performed with a NextSeq 500 system (Illumina; 150-bp paired-end configuration; Nextera XT Kit; DataSet 2) [22]. Raw reads (*n* = 7,941,390) were trimmed and filtered with AlienTrimmer v0.4.0 [24]. De novo assembly and annotation were performed with SPAdes (v3.12.0; –careful) and the NCBI Prokaryotic Genome Annotation Pipeline (PGAP v5.3; DataSet 3) [23, 25, 26]. Whole-genome sequence of ECC445 strain has a 95-fold coverage and BUSCO single-copy completeness of 98.60% (v5.3.2) [27]. It is 5,211,280-bp long divided onto 68 contigs, and presents a G + C content of 55.78% (QUAST v5.0.2; DataFile 3) [14, 28].

Whole-genome sequencing allowed accurate species identification using FastANI v1.3 and Genome-to-Genome Distance Calculator (GGDC; v3.0) against last *Enterobacter* references panel provided by Feng Y. and colleagues [3, 29–31]. Results confirmed that ECC445 strain is closely related to *E. chengduensis* with the novel Sequence Type ST1533 (Average Nucleotide Identity – ANI: 98.48%; in silico DNA-DNA Hybridization – isDDH *formula 2*: 88.20%) [5, 11, 29, 32, 33].

On June 29, 2022, a total of 19 *E. chengduensis* with complete metadata was retrieved from the NCBI database [13]. All these whole-genome sequences have passed our quality control (DataFile 4) [15]. To place ECC445 isolate in a global context, a core-genome alignment with Roary v3.13.0 was performed using reference sequences of *Enterobacter*, including all previous *E. chengduensis* (DataFile 5) [3, 13, 16, 34]. If only fastqs were available, de novo assembly was performed with SPAdes [25]. All additional sequences were annotated with PGAP as described above [26]. Maximum-likelihood phylogenetic reconstructions were performed by IQ-TREE (v2.1.2) and associated dependencies (*i.e.* ModelFinder and UFBoot2; –merit BIC: GTR + F + R8 –ufboot 1,000 –bnni; DataFile 6) [17, 35–37]. The tree was drawn with iTOL v6.4.2 (DataFile 7) [18, 38].

To date, ECC445 strain is the first *E. chengduensis* described in the natural environment and is a probable human pathogen (81.9%; PathogenFinder v1.1; DataFile 8) [19, 39]. The remaining 19 *E. chengduensis* isolates were recovered from human samples (*i.e.* ST414 *n* = 18; ST1535 *n* = 1). Most of them were described from China (10/19, 52.6%). Unlike ECC445, many of these strains had acquired resistance genes and plasmids (ResFinder v4.1, PlasmidFinder v2.1; details in the DataFile 4) [15, 40, 41].

The CRISPRCasFinder tool v1.1.2 identified a CRISPR/Cas system in the strain ECC445 genome (3 array sequences; Cas type I-F with evidence level of 4; DataFile 9) [20, 42]. As shown in DataFile 10 [21], none of the 46 CRISPR spacer sequences from strain ECC445 were found in the other *E. chengduensis* isolates using the CRISPR Comparison ToolKit (CCTK; v0.8.4) [43]. In contrast, some spacer arrays were identical between ST1535 and ST414 strains. These observations are consistent with the evolution of ECC445 ST1533 isolate in a different ecosystem than other *E. chengduensis* STs.

## Limitations

This Data note was limited to a single draft genome sequence. To overcome this limitation and place this strain in a global context, an extensive analysis of *E. chengduensis* strains was performed. Its correct identification by the hsp60 approach was confirmed here by whole-genome sequencing. However, further analysis on a larger collection is needed to appreciate the robustness of this technique to identify *E. chengduensis* among other ECC members.

## Data Availability

The genomic data described in this Data note can be freely and openly accessed on GenBank of NCBI. The partial hsp60 coding sequence of this *E. chengduensis* strain is available on OM687483 [10]. In addition, the SRA accession of the ECC445 strain is SRR16640525 [22], and the annotated assembly is version JAKLRZ000000000.1 [23]. Associated DataFiles are available on Figshare: antibiotic resistance profile and analysis of the sequence quality of the ECC445 strain [8, 14]; PathogenFinder and CrisprCasFinder [19, 20]; and global analysis of *E. chengduensis* from Genbank [15–18, 21]. The detailed methodology is available on the reference [9].

## Abbreviations

ANI: Average Nucleotide Identity
CCTK: CRISPR Comparison ToolKit
ECC: *Enterobacter cloacae* Complex
GGDC: Genome-to-Genome Distance Calculator
hsp60: Heat-shock protein 60
isDDH: in silico DNA-DNA Hybridization
PGAP: Prokaryotic Genome Annotation Pipeline
ST: Sequence Type
